# Guidelines for preclinical and early phase clinical assessment of novel radiosensitisers

**DOI:** 10.1038/bjc.2011.240

**Published:** 2011-07-19

**Authors:** K J Harrington, L J Billingham, T B Brunner, N G Burnet, C S Chan, P Hoskin, R I Mackay, T S Maughan, J Macdougall, W G McKenna, C M Nutting, A Oliver, R Plummer, I J Stratford, T Illidge, NCRI Clinical and Translational Radiotherapy Research Working Group

**Affiliations:** 1 The Institute of Cancer Research and Royal Marsden NHS Foundation Trust, Targeted Therapy Laboratory, Section of Cell and Molecular Biology, Chester Beatty Laboratories, 237 Fulham Road, London SW3 6JB, UK; 2 Cancer Research UK Clinical Trials Unit, University of Birmingham, Birmingham, UK; 3 Gray Institute for Radiation Oncology and Biology, University of Oxford, Oxford, UK; 4 Department of Oncology and Oncology Centre, Addenbrooke's Hospital, Cambridge, UK; 5 National Cancer Research Institute, London, UK; 6 Mount Vernon Hospital, Northwood, Middlesex, UK; 7 North Western Medical Physics, The Christie NHS Foundation Trust, Manchester, UK; 8 Cardiff University School of Medicine, Velindre Hospital, Whitchurch, Cardiff, UK; 9 Northern Institute for Cancer Research, University of Newcastle upon Tyne, Newcastle upon Tyne, UK; 10 University of Manchester, Manchester, UK; 11 School of Cancer, Enabling Sciences and Technology, University of Manchester and the Christie NHS Foundation Trust, Manchester, UK

**Keywords:** assessment, novel radiosensitisers, early, preclinical, clinical

## Summary

There is a growing appreciation of the potential value of combining novel molecularly-targeted drugs with radiotherapy or chemoradiotherapy. Such approaches have the potential to improve locoregional disease control and cure rates across a diverse range of tumour types. In this report, we outline a rational framework for developing novel drug–radiation combinations. In doing so, we make recommendations regarding the core preclinical data sets that are required to serve as justification for studies in humans and describe potential clinical trial designs that may be adopted by investigators.

Radiotherapy (RT) has a pivotal role in the management of many tumours, such that ∼50% of cancer patients will receive RT during the course of their illnesses and 40% of those cured of cancer will have received RT as part of their treatment (http://info.cancerresearchuk.org/). Indications for prescribing RT include (i) definitive, curative (radical) treatment; (ii) adjuvant therapy following surgery in an attempt to eradicate microscopic (or rarely macroscopic) residual disease; and (iii) as palliative treatment to ameliorate cancer-related symptoms. In the majority of situations, RT is a highly localised treatment that targets defined volumes of tissue that are known (or suspected) to contain cancer cells. Wide-field hemi-body (Bashir *et al*, 2008) or total body (Adkins and DiPersio, 2008) irradiation techniques are used rarely in very specific indications, such as metastatic bone disease or ‘conditioning’ before transplantation in haematological malignancies, respectively. In addition, the latest developments in stereotactic body RT (SBRT) techniques have also resulted in protocols that aim to treat oligometastatic disease spread through an organ (or more than one organ) with very precisely delivered RT fields and ablative doses (Benedict *et al*, 2010). This use of SBRT will certainly expand in the next decade and should be an active area for clinical trials.

In recent years, significant improvements have occurred in our technical ability to deliver RT to the target volume (tumour and/or locoregional lymph nodes), while limiting its delivery to critical normal structures. Techniques such as three-dimensional conformal RT, intensity-modulated RT and image-guided RT offer the prospect of significant benefits, both in terms of dose escalation within the tumour and dose sparing in normal organs (Bhide and Nutting, 2010; Staffurth, 2010). Clinical studies to test each of these strategies are ongoing in various tumour types; however, the expectation is that gains in tumour control probability may be clinically significant but relatively modest (<10%). In addition, in the near future it is likely that an increasing number of centres will have the capacity to deliver proton and heavy ion therapy. This technology offers highly conformal treatment and may allow clinicians to achieve dose escalation in the tumour while simultaneously sparing normal structures.

Systemic cytotoxic chemotherapy is increasingly combined with RT in an attempt to increase tumour control as well as targeting micrometastatic disease outside the radiation fields early in the treatment process (Bentzen *et al*, 2007). A range of cytotoxic agents has been tested in early trials, but most recent studies have focussed on platins, anti-metabolites (5-FU, gemcitabine) and taxanes. The data set derived from a meta-analysis in squamous cell cancer of the head and neck is particularly strong and definitively shows that the greatest benefit from combining chemotherapy with RT accrues when the agents are administered concomitantly (Pignon *et al*, 2000). Furthermore, the most clinically active drug is a DNA-damaging agent, cisplatin. These findings suggest that the temporal relationship between the two modalities and the mode of action of the cytotoxic drug are important components of the interaction and have led to cisplatin being used as a ‘radiosensitiser’ in a range of indications. In the case of radical and adjuvant RT, a number of studies have confirmed benefit when systemically administered cytotoxic chemotherapy is combined with RT (chemo-RT) (Pignon *et al*, 2000; Green *et al*, 2001; Urschel and Vasan, 2003; Bernier *et al*, 2004, 2005; Cooper *et al*, 2004; Lordick and Siewert, 2005; Auperin *et al*, 2006; Stupp *et al*, 2009). These studies across a range of tumour types (head and neck, lung, uterine cervical, gastric, oesophageal cancers and glioblastoma multiforme) have shown improved locoregional control, progression-free survival and, in some circumstances, overall survival. There is, as yet, little evidence that this systemic therapy has a strong influence on the rates of distant relapse. It is likely that this trend will continue and chemo-RT may become a standard of care in a wider range of cancers. In the context of palliative RT, there are currently no clinical scenarios in which combined drug and radiation therapy is the standard of care.

While researchers have been eager to exploit the benefits of concomitant chemo-RT to achieve better anti-tumour effects, any additional increase in tumour control appears in most clinical situations to be associated with an increase in acute and late normal tissue toxicity. There remain serious deficiencies in our understanding of the additional burden of acute and chronic side effects borne by the patients. However, it is clear that most current chemo-RT regimens are delivered close to (or even at) the limits of normal tissue tolerance, such that further treatment intensification by increasing the cytotoxic drug dose or by adding different classes of cytotoxics is not a viable strategy (Bentzen and Trotti, 2007; Machtay *et al*, 2008). In essence, the central problem lies with the lack of tumour specificity of cytotoxic chemotherapy such that normal tissues are also sensitised to the effects of RT. Therefore, in order to build on the benefits of chemo-RT, it will be necessary to develop agents that can selectively target cancer cells.

In recent years, by dissecting the molecular biological basis of cancer, we have begun to identify potential targets that may be manipulated to enhance the radiation response selectively in tumours. Some of these alterations are generic since they are present in a wide range of tumour types, while others are more specific and present only in a relatively narrow range of tumours. Hanahan and Weinberg (2000) provided a useful framework for categorising the steps in cancer development, progression, spread and response to treatment in terms of the so-called ‘hallmarks of cancer’ (Hanahan and Weinberg, 2000). Many of these traits can be invoked (singly or in combination) to explain the fundamental observations enshrined in the five Rs (repair, repopulation, redistribution, reoxygenation and radiosensitivity) of classical radiobiology (Harrington *et al*, 2007). Analysis of the ‘circuit diagrams’ of cell signalling pathways highlights points at which pharmacological intervention may have the potential to enhance the radiation response.

Pharmaceutical companies, ranging in size from multinational giants to small biotechnology outfits, and large academic institutions have established drug discovery programmes that have already introduced a large number of novel compounds into early phase clinical trials in a wide range of tumour types. In the majority of cases, the standard approach is to evaluate the compound as a single agent and then to consider combination approaches with existing licensed cytotoxic drugs. Such combination studies fit conveniently into standard phase I models in end-stage disease where treatment is palliative and toxicity end points are reached during the first one or two 3-week cycles of treatment (Yap *et al*, 2010). However, there is a growing appreciation that this strategy of drug development may neglect the opportunity to assess targeted drugs in the more challenging environment of clinical trials involving radiation. As will be discussed later in this paper, such studies may involve patients with newly diagnosed, potentially curable cancers and toxicity end points may be reached months (or even years) after completion of treatment. Despite these apparent difficulties, the significance of this therapeutic opportunity should not be underestimated by clinicians or pharmaceutical companies. Combining novel agents with established chemotherapeutic drugs in palliative treatment of solid cancers may lead to modest improvements in response rates, progression-free and overall survival. However, apart from a few relatively rare malignancies, such as gastrointestinal stromal sarcoma, there is currently little evidence that targeted drugs are able to induce high response rates that deliver durable disease control (remissions or stable disease) in the majority of solid cancers (Judson, 2010). In contrast to this palliative setting with low response rates, the clinical situation is very different where RT or chemo-RT are used with curative intent. In the latter setting, the addition of a novel agent may potentially lead to improved response rates that convert to improved overall survival and a greater number of long-term survivors.

For the development of novel therapies, financial considerations can be critical to success. Drug development is an expensive business and dictates that the cost of new agents is high in order to allow companies to recoup development expenditure and make a profit within the lifetime of the licence. When the improvements achieved by novel targeted drugs, either alone or combined with palliative chemotherapy, are subjected to cost-benefit analysis by organisations such as NICE in the United Kingdom, the new agents frequently fail to meet the required financial thresholds of cost per quality-adjusted life year and, thus, are not approved for use (http://www.nice.org.uk/nicemedia/pdf/TA172Guidance.pdf, http://www.nice.org.uk/newsroom/pressreleases/BevacizumabForTreatingMetastaticColorectalCancer.jsp). Perhaps this situation would occur less frequently if targeted drugs were used in a potentially curative setting with RT or chemo-RT, where the improvements in median overall survival may be sufficiently large to persuade regulators to approve the additional costs. It is also likely that the cost of drug development will fall appreciably in the next decade as trials become ‘smarter’ in their design. This factor may also encourage pharmaceutical companies to test their agents with radiation.

In this report, we seek to provide a rational framework for developing novel drugs that are capable of increasing tumour control when used in combination with RT. In doing so, we make recommendations regarding the core preclinical data sets that are required to serve as justification for studies in humans and outline potential clinical trial designs that may be adopted by investigators.

## Preclinical assessment of novel agents combined with RT

**Figure 1:**
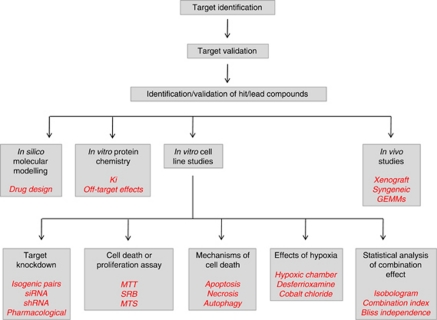
Summary of the components of a robust preclinical evaluation package for a putative targeted radiosensitiser. Abbreviations: Ki=dissociation constant; GEMMs=genetically engineered mouse models; siRNA=small interfering ribonucleic acid; shRNA=short hairpin ribonucleic acid; MTT=(dimethylthiazol-2-yl)-2,5diphenyltetrazolium bromide; SRB=sulforhodamine B; MTS=3-(4,5-dimethylthiazol-2-yl)-5-(3-carboxymethoxyphenyl)-2-(4-sulfophenyl)-2H-tetrazolium.

Conducting early phase clinical trials of novel agents in combination with RT represents a very significant regulatory, logistical, ethical and financial undertaking. Such clinical studies must be underpinned by a robust package of preclinical data in order to justify exposing patients to the risks of the treatment. In addition, clinicians will demand to see evidence that the combination is likely to be safe and has improved efficacy. Finally, drug development teams will need reassurance that treatment-related toxic events are unlikely to blight the future prospects of the study drug. Therefore, it is clear that the quality of the preclinical data set is most likely to determine whether, or not, a novel agent will enter clinical testing with RT. However, no consensus guidelines exist to direct preclinical testing of putative radiosensitisers. In this document, we seek to devise appropriate guidelines that will provide the core data sets necessary to support the development of novel radiosensitisers. Figure 1 summarises the key areas that must be considered in this process.

### Target identification/validation

Selection of therapeutic targets should have a sound mechanistic basis such that interference with the target has the potential to modulate key processes in cancer biology (Yap *et al*, 2010) and/or the radiation response (Zaidi *et al*, 2009; Mierzwa *et al*, 2010; Verheij *et al*, 2010). In fact, for most novel agents that have been developed to target cancer cells, it is possible to formulate a hypothesis whereby the agent may act as a radiosensitiser.

Since the ultimate goal of developing novel radiosensitisers is to improve clinical outcomes in patients with cancer, the first step in target identification should be to compile a compendium of molecular pathways that are known to have an important role in the biological behaviour of cancers that are routinely treated with RT. More importantly, particular emphasis should be given to pathways that are known directly to modulate the radiation response. By cross-referencing this list of pathways (and specific molecules on the pathways) with inventories of drugs that target them and are already in preclinical and clinical development, it should be possible to rank treatment approaches according to their relative significance and the likely timescale in which they will be achievable. A natural consequence of this process will be the identification of generic and specific targets: the former will apply to a broad range of tumour types (e.g., targeting the DNA damage response) (Helleday *et al*, 2008) while the latter may only be relevant to a small number of tumours (e.g., EGFR variant III in head and neck cancer and glioblastoma) (Sok *et al*, 2006; Mukherjee *et al*, 2009).

In order to avoid a piecemeal approach in which some targets are neglected while others are the subject of overlapping research by different groups, there is an urgent need to establish a network of research teams with expertise in molecular radiobiology and the desire to test the potential of targeted drugs to act as radiosensitisers (Maughan *et al*, 2010). Even with such an initiative, it must be understood that the number of possible targets and the availability of more than one drug for each target dictates the need for consensus guidelines that can be used to aid target selection and prioritisation, preliminary *in vitro* and *in vivo* testing and subsequent early phase clinical trials.

### Identification and validation of hit and lead compounds

#### Protein chemistry

If the crystal structure of the target protein has been solved, *in silico* molecular modelling studies should be available to guide design, synthesis and selection of potential inhibitors for *in vitro* assessment. Such modelling studies may also allow preliminary modification of the chemical material to improve its suitability for *in vivo* use and avoidance of toxicological outcomes (Workman, 2003a; Price *et al*, 2009). Initial *in vitro* analyses should include measurement of the dissociation constant (Ki) of the inhibitors from purified protein. This measure provides a standard for comparing potency and selectivity and should, ideally, be in the low nanomolar range. In addition, in order to screen for potential off-target effects, a range of related (and unrelated) protein targets should also be tested. These analyses should ensure that a limited number of compounds proceed to formal *in vitro* screening. In circumstances where the crystal structure has not been solved, it will be necessary to screen libraries of candidate molecules to test for activity (Workman, 2003a). These high-throughput screens allow the interaction between the target and the test agents to be tested in automated assays to identify a number of ‘hits’, which are chemical entities that can serve as the basis for further *in vitro* testing (see below) (Garrett *et al*, 2003; Eglen and Reisine, 2009).

#### Cell line studies

Selection of appropriate cell lines for *in vitro* analysis of the effects of targeted agents is a critical decision point. For those agents that are hypothesised to interact with targets that are aberrantly expressed in a wide range of cancers (i.e., generic targeting), selection of cell lines should be based on knowledge of expression of that target in individual tumour types with an eye on likely subsequent clinical evaluation. Many targeted compounds will already have been tested against the NCI-60 panel of cell lines (http://dtp.nci.nih.gov/docs/misc/common_files/cell_list.html), and such data may be useful in selecting appropriate cell lines.

For those targets that are relevant to a limited number of tumours (cell type-specific targeting), it may be more appropriate to test a panel of cell lines derived from that tumour type rather than a broad range of tumour cell lines. For example, the demonstration of improved outcomes in patients with head and neck cancers when EGFR targeting is combined with radiation will naturally lead to new EGFR-targeted therapies being assessed with radiation in head and neck cancer cell lines. Similarly, the emerging importance of Braf as a target in melanoma provides a strong rationale for testing new Braf inhibitors with radiation in melanoma cell lines. In recent years, there has been increasing attention paid to the importance of testing cell line identity, in view of suggestions that 20% of cell lines may be misidentified (UKCCCR Guidelines for the Use of Cell Lines in Cancer Research 2000; Masters *et al*, 2001; Cabrera *et al*, 2006). Therefore, in studies involving cell type-specific targeting, it is recommended that investigators identify their cell lines using readily available technologies such as short tandem repeat (STR) profiling (Masters *et al*, 2001).

*In vitro* testing of normal cells in an attempt to understand potential tumour selectivity and to predict possible normal tissue toxicity is an area that is fraught with difficulties. Many of the cell types that are relevant for studies of normal tissue toxicity will fail to grow well *in vitro* (and if they do so this may be a reflection of their divergence from their truly normal counterparts). Furthermore, for standard assays of radiosensitivity based on clonogenic survival, many of these cell types will fail to form colonies when plated at limiting dilutions. Therefore, such assays of cell death may fail to reflect the true radiosensitivity of normal tissues. Colorimetric assays of cell survival (dimethylthiazol-2-yl)-2,5diphenyltetrazolium bromide (MTT) (Carmichael *et al*, 1987), sulforhodamine B (SRB) (Skehan *et al*, 1990), 3-(4,5-dimethylthiazol-2-yl)-5-(3-carboxymethoxyphenyl)-2-(4-sulfophenyl)-2H-tetrazolium (MTS)) (Cory *et al*, 1991) can be used as alternative means of assessing radiation-induced cytotoxicity (or often, more correctly, cell proliferation). However, these assays do not provide a measure of clonogenic survival and, as yet, cannot be used to predict acute or late normal tissue toxicity. In the near future, there may be scope to model the effects of novel agents on normal tissue radiation responses through *in vitro* analysis of the kinetics of DNA repair (e.g., resolution of double-strand breaks measured by *γ*-H2AX foci or the formation of Rad51 foci) (Rakhorst *et al*, 2006; Sak and Stuschke, 2010; Redon *et al*, 2011).

*Demonstration of target knockdown:* Studies that show that the novel agent is capable of hitting the target molecule in relevant cell line systems are an absolute requirement of drug development (Workman, 2003b). Ideally, such studies should provide a number of lines of evidence to support the mechanistic basis of the targeted approach, including evidence that target knockdown is associated with modulation of the radiation response. There are a number of ways in which a target can be hit. These range from isogenic cell line pairs (Torrance *et al*, 2001), which differ only in the expression of the gene of interest, through to sequence-specific gene silencing using short interfering RNA or short hairpin ribonucleic acids (Harborth *et al*, 2001; Scherer and Rossi, 2003; Tiscornia *et al*, 2003) and small molecule or monoclonal antibody-mediated pharmacological modulation. Whatever method is used to interact with the target, there should exist robust, quality assured and validated assays to demonstrate the effect. Such assays may include quantitative reverse transcription PCR, western analysis for total and phospho-proteins, proteomic analysis, flow cytometry or ELISA to demonstrate changes at the RNA or protein levels.

*Measurement of cell death*: There exist a number of assays that can be used to measure cell proliferation or death. Colorimetric assays, such as MTT, SRB or MTS assays (Carmichael *et al*, 1987; Skehan *et al*, 1990; Cory *et al*, 1991), have the advantage of lending themselves to relatively rapid, high-throughput analysis in a simple 96-well format. However, as discussed above, many of these assays are more appropriately considered as measures of cell proliferation rather than cell survival and it is the latter issue that is fundamental to the development of new radiosensitisers.

While colorimetric assay formats can be invaluable as a means of selecting candidate molecules for further testing, any putative radiosensitiser should be formally assessed in a clonogenic cell survival assay, which remains the *in vitro* ‘gold standard’. This analysis will usually be based on single-fraction radiation doses across the range between 0 and 8 Gy. Although it may be attractive to have data on fractionated doses of radiation, the format of a standard clonogenic assay makes such studies difficult to perform. Experiments should be designed to allow derivation of dose or sensitiser enhancement ratios (DER or SER) (Hall *et al*, 1982; Lally *et al*, 2007), where DER equals the surviving fraction at an indicated radiation dose divided by the surviving fraction at the same dose of radiation plus the potential sensitiser, with appropriate account taken of plating efficiency. Even relatively low DER/SER values (range 1.2–1.5) may be indicative of a useful effect, especially if they are derived at clinically relevant radiation doses (e.g., 2 Gy single fraction).

*Mechanism of death*: The numbers of recognised types of tumour cell death continue to increase with new biological insights. Reports have been published demonstrating that radiation can kill cancer cells through apoptosis, mitotic catastrophe and autophagy or can induce terminal growth arrest senescence (reviewed in Eriksson and Stigbrand, 2010; Thoms and Bristow, 2010), depending on the cell line, the combination treatment and the experimental design. In fact, there is a paucity of data in the literature to demonstrate that *in vitro* experience with radiation and/or drug treatments is replicated *in vivo*. Nonetheless, it would appear reasonable to expect data to be available on the type of cell death that occurs when tumour cells are treated with radiation, drug treatment or the combination. Standard assays for apoptosis (flow cytometry, western analysis for PARP or pro-caspase-3 cleavage), mitotic catastrophe (micronucleus, >4 N ploidy) and autophagy (confocal microscopy for acidic vacuolar organelles, flow cytometry for acridine orange and western analysis for LC3I/II) are all appropriate measures of the cellular effects of the combination treatments.

*Effect of targeted agent in normoxic and hypoxic conditions*: Given the fact that tumour hypoxic is a major determinant of the radiation response *in vivo* (Overgaard and Horsman, 1996), the experiments discussed in sections above should also be repeated under hypoxic conditions. These analyses should be conducted using hypoxic chambers rather than pharmacological means of mimicking hypoxia.

*Analysis of combination therapy*: *In vitro* analyses of the interactions between RT and novel radiosensitisers should be based on formal statistical tests – such as isobolographic analysis (Steel and Peckham, 1979), combination index (CI) analysis (Chou and Talalay, 1984) or Bliss independence analysis (Bliss, 1956). Isobolographic analysis is an approach that represents zero-interaction curves of two agents and fits data from experiments in which they are combined in order to determine if they show additive, sub-additive or supra-additive (synergistic) interactions. Classic isobolographic analysis has been perceived as resource intensive and, therefore, it has not been widely adopted (Niyazi and Belka, 2006). CI analysis has become increasingly popular in recent years, but it was initially designed for testing interactions between combinations of drugs. The methodology is based on initial definition of the concentration that inhibits growth by 50% (the IC_50_ or GI_50_) and then combining fixed ratios of the IC_50_ values according to a checkerboard design (e.g., 0.25/0.25; 0.5/0.5; 1.0/1.0; 2.0/2.0; 4.0/4.0) (Chou and Talalay, 1984). The IC_50_ is generally derived from MTT/SRB/MTS colorimetric assay and this same methodology is used for the drug combinations. Under these circumstances, a CI of 0.9–1.1 denotes an additive interaction, >1.1 denotes antagonism, and <0.9 denotes synergy. However, when using this approach with radiation, the degree of cell kill seen with multiples (e.g., two- or four-fold) of the IC_50_ frequently result in 100% cell kill and such effects do not lend themselves to CI analysis. Instead, experiments can be done using a range of doses in a non-constant ratio checkerboard design in order to derive a CI (Twigger *et al*, 2008). Bliss independence analysis is an alternative methodology in which observed effects (*F*_obs_) are compared with expected effects (*F*_exp_). For example, when two agents are combined at IC_50_ doses, the expected cell kill (*F*_exp_) is 75% if the agents act independently (50% of cells are killed by one agent and 50% of the remaining cells (i.e., 25%) are killed by the other agent). The Bliss equations define the following conditions: if *F*_obs_−*F*_exp_=0, the interaction is defined as independent (or additive); if *F*_obs_−*F*_exp_>0, the interaction is greater-than-additive (or synergistic by convention); if *F*_obs_−*F*_exp_<0, the interaction is less-than-additive (or antagonistic by convention).

In the future, it will become increasingly important that the interactions of triple therapy combinations (radiation, cytotoxic drug (e.g., cisplatin) and novel radiosensitiser) can be assessed. An elegant methodology for adapting isobolographic analysis to triple combinations has been described by Niyazi and Belka (2006).

## *In vivo* analysis

A number of issues need to be considered when designing *in vivo* studies of putative radiosensitisers. The vast majority of studies will be conducted in rodent models and, for these purposes, mice are more likely to be used than rats for reasons of cost and availability. Most assessments will inevitably involve evaluation of human xenograft tumours in immunocompromised mice (Rygaard and Povlsen, 1969). Athymic nude mice are preferable to other immunocompromised murine models, such as SCID or NOD-SCID mice that have coexisting abnormalities of DNA repair, which are likely to influence normal tissue toxicity (Chang *et al*, 1993). The perceived advantage of using xenograft tumours in nude mice is that researchers are able to study the effects of therapy directly on human tumours of known histological subtypes. This view must be counterbalanced by the knowledge that many common studies of human xenograft tumours have been maintained in the laboratory for many years, or even decades, and may not accurately reflect the biology of the parental tumour. Indeed, in recent years, it has become clear that a number of cell stocks have become contaminated or supplanted. Many journals now require genetic characterisation of cell lines using STR assays as part of the review process(http://www.aacr.org/home/scientists/publications-of-the-aacr/author-services-center/cell-line-authentication-information.aspx). An alternative to using established cell lines involves taking tumour material directly from patients and implanting it in immunodeficient mice (Morgan *et al*, 2010). Such studies allow researchers to work with cells that have not undergone *in vitro* genetic drift and the presence of tumour stroma (at least in the short term) may add some value to these experiments. It is likely that these sorts of experiments will become more popular in the near future.

An alternative to implanting human tumours in immunodeficient mice is to generate counterpart murine tumours *in situ* in relevant organ systems in immunocompetent mice. There are three approaches to this: spontaneously arising murine tumours; carcinogen-induced tumours; and genetically engineered mouse models (GEMMs) (Politi and Pao, 2011). GEMMs are generated by transforming mouse cells by introducing specific genetic lesions in a manner that can be controlled spatially (tissue specificity) and temporally (ability to switch-on and -off the effect). These systems are proving to be extremely powerful tools for studying the biological processes that underlie cancer formation and progression, but are also assuming greater importance as models in which anti-cancer therapies can be tested. For example, Becher *et al* (2010) have reported the effects of radiation plus perifosine (an agent that modulates AKT signalling) in a genetically and histologically accurate model of brainstem glioma induced by overexpressing platelet-derived growth factor receptor *α* in the posterior fossa of neonatal mice (Becher *et al*, 2010).

Most preclinical reports of the effects of RT in murine models have been based on subcutaneous or intramuscular xenograft tumours. Irradiating tumours in these locations (where irradiation of normal tissues can be minimised) provides a means of measuring tumour growth delay or resolution, but gives little or no information on the toxicity of the combination. Even assessment of cutaneous toxicity of the combined treatment is of limited value, since murine skin is not a good model for human skin. New developments in the use of small animal microirradiation units are likely to have a significant impact on this field of study. It is now possible to model conformal clinical RT in animal models using state-of-the-art image-guided irradiation of tumours growing in orthotopic sites (Saha *et al*, 2010; Clarkson *et al*, 2011). Such studies have the potential to provide useful information on both tumour growth delay/control and normal tissue toxicity.

In addition, there are emerging data that show that some of the anti-tumour effects of radiation may be mediated by effects on the tumour stroma and by the immune system (Formenti and Demaria, 2009). Clearly, using xenograft tumours in nude mice provides no opportunity for studying immune-mediated effects. Therefore, there may be a role for use of immune competent mice bearing syngeneic murine tumours in certain circumstances, but concerns must exist about the validity of data derived from these studies.

Therefore, as discussed above, the choice of the most appropriate animal model for combination radiation studies is likely to be an area in which there will be considerable change in the next decade. However, for the present, it is likely that studies on xenograft tumours in nude mice will continue to dominate the landscape.

The main questions relating to the conduct of *in vivo* studies are as follows:

### How many cell lines need to be tested using *in vivo* studies?

If the novel agent is thought to be a generic radiosensitiser, *in vivo* studies should include a panel of cell lines selected from the major tumour types that are treated by radiation. This selection should also reflect the tumour types that are likely to be represented in future phase I studies (typically lung, breast, prostate, head and neck, colorectal, glioma, melanoma). For tumour type-specific agents, it would seem reasonable to recommend that *in vivo* studies include a minimum of at least two separate tumour cell lines of that histotype.

### What radiation dose schedule should be tested?

Many studies that look for radiosensitisation are based on RT dose and fractionation regimens, which do not accurately reflect clinical practice. For proof-of-principle studies to demonstrate that a particular agent is able to enhance the effect of RT *in vivo*, it is perfectly reasonable to use abbreviated dose schedules. Therefore, single doses between 2 and 8 Gy or short-course fractionation regimens (e.g., from 9 Gy in three fractions to 20 Gy in five fractions) are entirely appropriate for these purposes. However, in order to get a clearer indication of the activity/toxicity of an agent, it may be important to conduct more protracted dosing studies in which daily radiation doses of 2 Gy are administered with repeated dosing of the radiosensitiser (see below).

### Which efficacy end point should be selected?

Therapeutic efficacy studies should only be performed once evidence has been obtained that the drug has a suitable pharmacokinetic profile to allow it to reach active concentrations within the tumour tissue. This latter issue can be confirmed by demonstrating modulation of the target (or its downstream signalling components) in tumour biopsies. Such studies of drug-on-target effects may also provide useful guidance on the most appropriate radiation schedule for combination experiments. Many studies in the literature report differences in tumour growth delay as evidence of an effect of a radiosensitiser. Typically, data are reported as the median time taken to reach a certain tumour volume (often a multiple of the original volume on the first day of therapy or the time taken to reach a specific maximum diameter (1.0–1.5 cm)). If the appropriate single agent controls (RT alone, drug alone) are included in such studies, they can serve as useful indicators of activity. However, if a number of agents are to be tested to select which one has the greatest SER and, hence, the greatest potential for clinical development, it may be preferable to use methodology that permits researchers to derive the tumour control dose-50 (TCD50) (Stone and Withers, 1975; Baumann *et al*, 1992). The TCD50 is the radiation dose delivered at 2 Gy per fraction that provides tumour control in 50% of tumours. Such studies may involve mirroring clinical dose schedules (30–35 fractions over 6–7 weeks) and, as a result, they are both labour intensive and expensive. Therefore, their use cannot be recommended as a routine for most putative radiosensitisers.

### Which toxicity end point should be studied?

Most *in vivo* models of tumour efficacy do not include a formal analysis of normal tissue toxicity. Certainly, most *in vivo* systems for irradiating xenograft (or syngeneic) tumours use implants in the flank (subcutaneous) or leg (subcutaneous or intramuscular) where important potentially dose-limiting normal organs will receive minimal doses of radiation. The use of orthotopic tumour models (e.g., intracerebral glioma, lung, pancreatic and head and neck cancer (Gridley *et al*, 2002; Epperly *et al*, 2010; Lee *et al*, 2010; Saha *et al*, 2010)) allows investigators to study effects on normal tissues, but these studies are usually geared towards efficacy end points. There are validated animal models for testing normal tissue toxicities, such as the intestinal crypt cell colony assay (Hornsey and Vatistas, 1963; Hagemann *et al*, 1971a, 1971b) or the ventral tongue mucosal ulceration assay (Dörr *et al*, 2002), but their use is restricted to a limited number of centres. An appropriate approach to the use of these models would include the recommendation that at least one of these systems is used in evaluating drugs that target the DNA damage response. This guidance recognises the severe phenotypes of normal tissue damage that occur in patients with syndromes involving DNA repair deficiencies (Pollard and Gatti, 2009). A key ‘go/no-go’ step in this assessment will be an evaluation of the relative degree of sensitisation (as measured by SER) of tumour *vs* normal tissues. Agents with a normal tissue SER that is greater than or equal to the tumour SER should not proceed to clinical development. On the other hand, the importance of measuring normal tissue toxicity in animal models is unclear for agents that target ‘oncogene addiction’ in cancers (e.g., mutation or overexpression of growth factor receptors or downstream signal transduction pathways). In such cases, animal studies should be considered optional with the alternative of incorporating them into the phase I analysis in patients.

## Clinical evaluation

The conduct of phase I studies of novel putative radiosensitisers with RT represents an important opportunity to improve locoregional tumour control for a range of tumour types. In addition, for the pharmaceutical companies and academic institutions responsible for drug development, this approach provides the best prospect for many of their agents to contribute to improving cure rates of common solid cancers. In order to achieve success with this goal, we must address a number of specific challenges that do not exist in standard phase I studies of systemic cytotoxic chemotherapy. These can be summarised as follows:

1. In phase I studies of a new agent combined with cytotoxic chemotherapy, both drugs are administered systemically such that all of the normal organ systems of the body are exposed to both agents and can be assessed for toxicity. Therefore, the tumour type and site are generally irrelevant when considering toxicity end points. In contrast, in a comparable group of patients with end-stage cancer who are to receive palliative RT, the site of the disease and its location relative to the body surface (superficial *vs* deep) determines which normal organs are exposed to the combination treatment. Therefore, in a typical group of phase I patients with a variety of tumour sites (e.g., cutaneous/subcutaneous disease, lung, bone or nodal metastases), it will not be possible to ensure that all normal organ systems are assessed in all, or any, of the three patients in each dose level. As a result, toxicity evaluation and decisions to escalate to the next dose may be based on incomplete data.
2. On the basis that combination studies may be difficult to design and perform in patients with end-stage disease, it appears reasonable to conduct such studies in patients with newly diagnosed, potentially curable cancers. However, in this setting, the risks may be perceived as being high. Any dose-limiting toxicity that causes an interruption in the delivery of RT is likely to have a negative impact on the probability of tumour control (Bese *et al*, 2007). For that reason, the ethical considerations around conducting such studies will need particular attention such that groups with historically poor treatment outcomes (e.g., locally advanced lung cancer, pancreatic cancer) are targeted. Further complexity is added by the fact that most curative regimens for solid tumours involve the use of concomitant chemo-RT. Adding novel targeted drugs to chemoradiation will require additional preclinical evaluation of the triple therapy, over and above that conducted for the radiation-novel drug combination.
3. Phase I studies are classically set up to detect acute treatment-related toxicities with a view to detecting dose-limiting toxicities and to recommend a dose for subsequent phase II testing (LoRusso *et al*, 2010). The relevance of this approach to phase I studies involving combinations of targeted drugs with radiation must be carefully considered. Dose escalation decisions are classically based on a 3 + 3 design in which the occurrence of an acute DLT in one of three patients in a cohort triggers recruitment of a further three patients to that dose level. If one of these additional patients also experiences a DLT, dose escalation is discontinued and the maximum tolerated dose is defined as the preceding dose level. In many clinical scenarios in which RT is used with curative intent, it is considered routine to encounter acute grade 3 toxicities when treating with RT alone. Therefore, it may be difficult to decide whether or not a toxic event occurring during RT is greater than might be expected and worthy of consideration as a DLT. Careful design of study protocols, including precise definition of reactions that are to be considered as a DLT, by experienced clinicians will be a key means of overcoming this potential hurdle.
4. In studies involving combining radical RT with novel drugs, the period of time over which acute toxicity end points have to be assessed will be much longer than in conventional studies of new agents combined with cytotoxic chemotherapy. For example, a course of RT may be delivered over 7 weeks. Unless there is a dramatic exacerbation of acute toxicity, its occurrence (often grade 3 as discussed above) may not allow a conclusion to be drawn about the safety of the combination. Instead, it may be necessary to observe the rate of resolution of the acute toxicity and use this as an index of safety and tolerability (Harrington *et al*, 2009). The recovery period is frequently 4–6 weeks. Thus, a patient may only be assessable for the toxicity end point 3 months after entering the study. Therefore, there will be long gaps between dose escalation decisions and the entire study is likely to be protracted. Novel clinical trial designs, such as the continuous reassessment method (Desai *et al*, 2007) or adaptive dose finding (Potter, 2002), should be used more frequently as a means of ensuring rapid recruitment and completion of studies.
5. Standard phase I methodology will completely fail to capture late toxic effects, even though such events might limit the applicability of the new agent in combination with radiation. Indeed, if dose escalation phase I studies were designed to study late toxicity end points, it would be necessary to follow successive cohorts for 6–12 months before being able to make dose escalation decisions (Guerrero Urbano *et al*, 2007a; Urbano *et al*, 2007b). Clearly, this is not a practical proposition. Instead, it will be necessary to design phase II and III studies that allow collection of long-term toxicity data.

### Phase I studies in patients receiving palliative RT

These studies can be difficult to design and complete, but the opportunities that they provide in terms of defining toxicity, efficacy and biological end points means that these studies should be a key component of the development of novel radiosensitisers. A number of factors need to be considered when designing studies for clinical scenarios in which palliative RT is delivered.

#### Palliative RT to bone metastases

The anatomical sites where palliative RT to bone metastases is delivered can be extremely varied and this factor has previously been a disincentive to include these patients in clinical trials of RT plus novel radiosensitisers. For example, palliative RT to a bone lesion involving a distal long bone (e.g., femur or humerus) is unlikely to cause acute toxicity to any normal tissue other than the skin. In contrast, irradiation of a lumbar or thoracic vertebral metastasis may also expose structures such as spinal cord/cauda equina, bowel, liver, stomach, oesophagus and lung to the dual effects of RT and the novel agents. However, the relatively large number of patients treated with RT for bone metastases (Agarawal *et al*, 2006) means that it should be possible to select homogeneous patient groups to provide meaningful toxicity end points from this population. In addition, pain scores and analgesic consumption can be used as surrogates of response (Lutz *et al*, 2009). The difficulties in obtaining serial biopsies (pre- and post-treatment) from patients with bone metastases and the methodological issues inherent in deriving pharmacodynamic (PD) data from such samples has also been seen as a disadvantage of studying this group of patients. However, the use of modern techniques (serum makers of bone damage, circulating tumour cells, functional imaging) may provide non-invasive means of deriving efficacy data from these studies (Tan *et al*, 2009; Costelloe *et al*, 2010; Padhani and Koh, 2011). The total radiation dose and fractionation regimen is also an important area to consider in such study design. There are, for example, clinical indications for using a range of fractionation schedules (Agarawal *et al*, 2006) – from single doses to 10 fractions over 2 weeks – and this provides an attractive opportunity to vary the number of fractions of radiation that are sensitised by a novel agent.

#### Whole brain RT

This approach has a number of potential advantages. First, there are a large number of patients with brain metastases (35% of patients with cancer) and there are few clinical trials for such patients. Therefore, studies including such patients with defined populations of solid cancers are likely to recruit rapidly due to a lack of competing interests. Second, the radiation technique can be standardised such that the tissues irradiated in all patients can be the same. This will mean that the toxicity end points are more likely to be valid, but this must be considered against the fact that there are few clinically significant rapidly dividing tissues in the irradiated volume. This will significantly limit the applicability of data to other body sites. The major disadvantages of treating patients with brain metastases are the overall very poor prognosis (and resulting limited toxicity data set that will be available), the fact that toxicity measures may be influenced by disease-related events outside the brain and the inability to obtain tissue samples pre- and post-treatment for PD analyses (Rao *et al*, 2007). Many of these concerns may be overcome by using functional imaging techniques (DCE-MRI, DW-MRI and PET) to provide non-invasive PD measures.

#### Palliative lung RT

This situation represents a very favourable indication for study. There are large numbers of patients treated with palliative lung RT for a number of histological tumour subtypes. The radiation technique can be adapted to allow evaluation of different important normal tissue effects. For example, irradiation of lateralised tumours away from the mediastinum will allow assessment of skin and lung toxicity, while irradiation of centralised tumours will allow assessment of acute mucosal (oesophageal) toxicity. A disadvantage of this indication is the fact that many patients are treated with single-fraction RT, thus limiting options for exploring effects of more protracted drug exposure (Lester *et al*, 2006). However, there are well-described fractionated regimens that would serve as very useful therapeutic models in which variable numbers of treatment fractions could be delivered concomitantly with a putative radiosensitiser (reviewed in Lester *et al*, 2006). Again, tumour biopsies for PD markers are not straightforward in this group of patients, but modern bronchoscopic techniques are likely to make such biomarker studies increasingly achievable.

#### Palliative RT to superficial or surface nodal or cutaneous metastases

Relatively large numbers of patients with a range of histological diagnoses are treated for tumour deposits at or close to the skin surface. Such disease is amenable to localised electron RT (thus limiting normal tissue irradiation) or megavoltage irradiation (encompassing a larger volume of normal tissue). In addition, serial biopsy will frequently be possible to provide PD biomarker data. Disadvantages of this scenario include the potential heterogeneity of irradiated sites (which will limit the validity of toxicity data) and the variability of histological subtypes (which may limit value of biomarker data). However, the large numbers of patients treated for this indication mean that recruitment of homogeneous groups should be possible in either single- or multi-centre studies.

### Phase I studies in patients receiving radical RT

Although conducting phase I studies of novel radiosensitisers in patients receiving palliative RT will be extremely valuable, such trials are unlikely to form the basis of registration studies that achieve FDA approvals. Instead, they are likely to provide important safety and mechanistic data that underpin definitive studies in patients treated with curative intent. Indeed, as discussed above, in a number of disease settings, the novel agent is most likely to be evaluated as part of a regimen involving concomitant chemo-RT. In this regard, specific methodological problems arise that must be addressed in the study design. The most important consideration is the need to avoid delays in the delivery of RT since this can have serious effects on the probability of achieving tumour control (Bese *et al*, 2007). In the worst case scenario, a toxicity related to the investigational medicinal product (IMP) may result in early termination of RT before a curative dose has been delivered, thus denying the patient the chance of tumour control. Alternatively, in patients receiving RT as part of an organ-preservation protocol, premature curtailment of the course of RT may dictate that the patient has to undergo mutilating ablative surgery (with all the consequent effects on quality of life). Such risks have to be balanced against the huge potential benefits of developing new therapies that are able to improve locoregional control, progression-free and overall survival rates. Careful implementation of clinical programmes based on a sound preclinical data set and, where appropriate, early phase studies in a palliative setting should minimise these risks and reassure study sponsors and regulatory agencies.

In order to integrate novel agents alongside RT, there are a number of possible study designs that should be considered. These alternatives will be presented and their respective strengths and weaknesses will be considered. In addition, an attempt will be made to consider the level of risk that the approach poses to the aim of delivering the full dose of radical RT.

#### Neoadjuvant or window of opportunity studies

**Figure 2:**
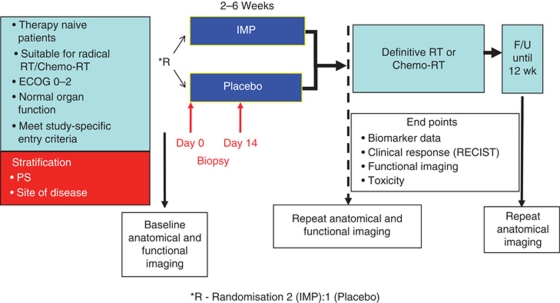
Typical phase 0/window of opportunity clinical trial design. Patients are randomised between the investigational medicinal product (IMP) and an inactive placebo for a variable time period (depending on the clinical situation governing management of the primary tumour). These studies have the potential to provide toxicity, response and biomarker (tissue and radiological) end points.

The period during which RT is planned represents a potentially valuable opportunity to study the biological activity of a novel agent. In most centres in the United Kingdom, the interval between the initial treatment decision and the delivery of the first fraction of RT is at least 2–4 weeks. Therefore, studies can be designed such that patients receive the study drug for a number of weeks before starting RT. Indeed, it is also possible to conduct randomised window of opportunity studies where patients receive either the IMP or a placebo. Patients discontinued study medication immediately before starting RT. By performing tumour and/or normal tissue biopsies before and after drug administration, useful biomarker data can be generated. In addition, inclusion of standard anatomical (CT, MRI) and functional (perfusion CT, dynamic contrast-enhanced or diffusion-weighted MRI, PET or PET-CT) imaging studies can provide data on tumour responses and PD outcomes from single agent therapy. An example of a window of opportunity study design is shown in Figure 2 (Del Campo *et al*, 2000).

This sort of clinical trial design is likely to be seen as low risk by regulators and pharmaceutical companies and has the obvious attraction of providing biomarker data from treatment-naive tumours. In addition, patients frequently view the treatment planning period as a ‘delay’ and are eager to commence treatment as soon as possible. Therefore, they are likely to be receptive to the notion of window of opportunity studies, even if they involve randomisation to a placebo arm, since they will perceive this as starting some form of therapy. By designing the study to include analyses of response, locoregional control and progression-free survival, it may also be possible to generate hypotheses about the potential interactions between the IMP and radiation. For example, in studies involving drugs that modulate angiogenesis, it is reasonable to hypothesise that vascular normalisation before starting RT may be associated with a better treatment outcome (Jain, 2005). Similarly, anti-proliferative drugs (such as growth factor receptor antagonists) may prime the tumour for a lower level of repopulation during RT and, thus, lead to improved treatment outcomes (Del Campo *et al*, 2000; Harrington *et al*, 2007).

However attractive a window of opportunity study might seem, it has the major disadvantage of providing no information on the effect of combining the IMP with RT. As such, these studies can only provide initial proof-of-principle data and, perhaps, some hints about potential radiosensitising effects. Ultimately, however, it will be necessary to perform studies in which the study agent is administered concomitantly with radiation.

#### Drug dose escalation studies

**Figure 3:**
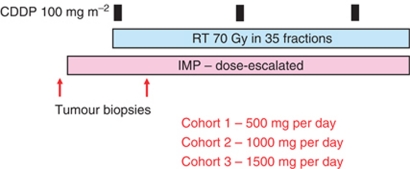
Drug dose escalation phase I trial design based on a clinical protocol in which lapatinib was added to standard chemoradiation in patients with stage III/IV head and neck cancer (Harrington *et al*, 2009). Patients were recruited in cohorts of three to each dose level. In the event of the occurrence of a predefined dose-limiting toxicity, an additional three patients were recruited to that dose cohort (classical 3+3 design). Subsequent dose escalation was only permitted if none of the additional three patients suffered a DLT. Optional tumour biopsies may be incorporated into these study designs to provide biomarker data.

In line with classical phase I methodology, this type of study involves treating cohorts of patients with a standard radiation dose in combination with gradually increasing doses of the IMP that are administered for the whole duration of the course of RT (Figure 3) (Harrington *et al*, 2009). The initial dose level of the IMP will have been determined in prior single agent studies and the frequency of dosing will be based on the pharmacokinetics of the drug. The choice of dose escalation scheme is important and may involve standard (e.g., cohort 1–100 mg per day, cohort 2–200 mg per day, cohort 3–300 mg per day, etc) or multiplicative (e.g., cohort 1–100 mg per day, cohort 2–200 mg per day, cohort 3–400 mg per day, etc) dose increments. The former will be appropriate for many agents (especially where there is a solid body of data for the drug as a single agent or in combination with cytotoxic chemotherapy). The latter should be considered risky in the context of radical RT where the occurrence of dose-limiting toxicity may have serious consequences for the chance of achieving local control. Alternatively, a modified Fibonacci design may be seen as a safe approach, especially where the drug has been shown to be a potent radiosensitiser in *in vitro* studies. Such designs use dose increments that become progressively smaller with ascending dose cohorts and, so, tend to mitigate against the risk of encountering unexpected severe dose-limiting toxicities that may interrupt treatment. Where possible, data from previous studies should be used to guide the dose escalation scheme and to minimise the number of cohorts so that the study can be completed in a timely manner.

The overall aim of this type of study is to keep the drug on the target throughout the course of treatment and to sensitise every fraction of RT. If possible, tumour/normal tissue biopsies should be obtained before and after starting treatment to study PD end points. Again, functional imaging studies may provide important information, especially in situations where repeated biopsies may be technically difficult or hazardous to the patient.

#### Drug duration escalation studies

**Figure 4:**
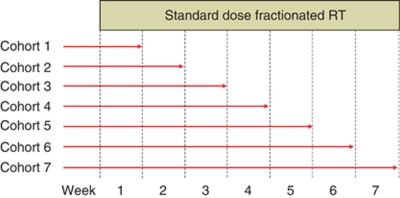
Drug duration escalation study. Successive patient cohorts are exposed to a predetermined dose of the study drug for an increasing number of radiation fractions.

In these studies, the objective is to escalate the total number of fractions of RT that are potentially sensitised by the drug (Figure 4). Therefore, a standard drug dose is administered throughout the study, but successive cohorts of patients receive the drug and radiation concomitantly for progressively longer periods of time. The drug dose that is used may be based on a prior level that has been shown to be tolerable or on an optimal biological dose that achieves maximal inhibition of its target. For standard radical courses of RT, which usually involve 5–7 weeks of treatment, the number of dose escalation cohorts should be chosen so that the study can be completed as rapidly as possible. Ideally, there should be no more than 4–5 cohorts.

**Figure 5:**
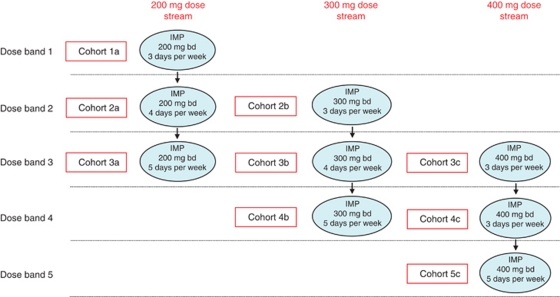
An example of a study involving continuous reassessment methodology. Patients receive standard radical RT (e.g., 70 Gy in 35 fractions) and receive the IMP according to predefined dose streams and within dose bands. This design allows for simultaneous evaluation of different doses and durations of exposure to the IMP.

The ultimate goal of this trial design is to escalate towards combining the drug and radiation for the maximum number of fractions (i.e., the entire course). Alternatively, it may only be possible to use the drug for part of the treatment course. In this regard, continuous reassessment methodology may be particularly useful as a means of escalating drug dose and drug exposure within the same study (Figure 5) (LoRusso *et al*, 2010). This approach has significant advantages in terms of completing studies in a timely manner, but may pose some problems by defining more than one maximum tolerated dose or recommended dose for phase II evaluation. Again, as with previous designs, obtaining biopsy material and performing functional imaging should be a priority in order to maximise the yield of PD data and, thus, shape subsequent study design.

#### Flip-flop studies

**Figure 6:**
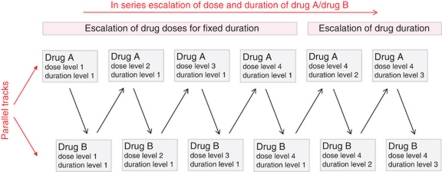
Flip-flop study design. Patients are recruited to two parallel streams in which different novels agents (Drug A and Drug B) are combined with radiation. Closure of a cohort in the Drug A study track triggers opening of recruitment to the Drug B study track (and *vice versa*). This design avoids gaps in patient recruitment by ensuring that recruitment to either track is always open.

By their very nature, phase I studies can be relatively slow to complete recruitment – not least because of the requirement to wait an appropriate length of time within and between study cohorts in order to allow assessment of the occurrence and resolution of toxicities. As a result, there are frequent intermissions during which patients cannot enter the study. An imaginative approach to this tendency for gaps in active recruitment is to conduct parallel studies in which different novel agents are assessed. As shown in Figure 6, eligible patients can be recruited to receive Drug A in combination with RT. When this dose cohort is full and patients are being followed up for toxicity, eligible patients are able to enter a parallel study of Drug B. Successive cohorts flip-flop between the parallel study tracks with the effect that uninterrupted recruitment can proceed with the generation of data on two different targeted drugs (http://www.cancerhelp.org.uk/trials/a-study-cediranib-azd2171-and-azd6244-with-chemotherapy-radiotherapy-rectal-cancer-dream). In addition, this design has the flexibility to allow escalations of both drug dose and the duration of drug exposure (see Figure 6). It is likely that this study design will become increasingly important as a means of maximising patient recruitment rates.

## Conclusions

Rapid developments in our understanding of cancer biology have yielded a huge number of potential therapeutic targets and, for many of these, novel agents have already been discovered or synthesised. The conventional approach to drug development has usually been based around testing these agents first on their own and then in combination with standard cytotoxic agents. Historically, even when there was a strong theoretical rationale for combination with RT, there was reluctance to undertake such studies among drug developers because the path from the laboratory to the clinic was seen as difficult and associated with additional perceived risks. This situation has begun to change in recent years with pharmaceutical and biotechnology companies becoming far more receptive to the potential benefits of combining their drugs with radiation. In order to accelerate this process, we have drawn up consensus guidelines that should provide a framework for preclinical and clinical development of novel radiosensitisers.
